# Study on the changes of CT texture parameters before and after HCC treatment in the efficacy evaluation and survival predication of patients with HCC

**DOI:** 10.3389/fonc.2022.957737

**Published:** 2022-10-28

**Authors:** Wei Zhou, Yinzhang Lv, Xuemei Hu, Yan Luo, Jiali Li, Haidan Zhu, Yucheng Hai

**Affiliations:** ^1^ Department of Radiology, Tongji Hospital, Tongji Medical College, Huazhong University of Science and Technology, Wuhan, China; ^2^ Department of Radiology, Lanzhou University Second Hospital, Lanzhou, China

**Keywords:** texture analysis, efficacy evaluation, grayscale histogram, TACE, hepatocellular carcinoma

## Abstract

**Objective:**

To investigate texture parameters of contrast-enhanced computed tomography (CT) images before and after transarterial chemoembolization (TACE) as a tool for assessing the therapeutic response and survival predication in hepatocellular carcinoma (HCC).

**Materials and methods:**

Data of 77 HCC patients who underwent three-phase dynamic contrast-enhanced CT examination within 4 weeks before and 4–8 weeks after TACE were collected and efficacy evaluation was performed according to the modified Response Evaluation Criteria in Solid Tumors (mRECIST) standard. The remission group consisted of 31 patients (12 with complete remission+19 with partial remission), while the non-remission group consisted of 46 patients (27 with stable disease+19 with progressive disease). Full-volume manual delineation of the region of interest (ROI) and texture analysis of the ROI were performed on the CT images using FireVoxel software. Changes in the 48 texture parameters from three-phase CT images before and after TACE were calculated and compared between the two groups. The receiver operating characteristic (ROC) curve and the areas under the curve (AUC) were used to analyze the diagnostic performance of texture parameters. A multifactorial Cox model was used for predicting survival. The C-indices of texture parameter difference values with predictive value, texture features model, and texture features combined with mRECIST in predicting OS were compared with those of mRECIST.

**Results:**

A total of 41 changes in texture parameters were statistically significant between the remission and non-remission groups. The receiver operating characteristic (ROC) curve showed that the AUC of changes in the 90th percentile in the arterial phase was the largest at 0.842. When the cut-off value was 70.50, the Youden index was the largest (0.621), and the sensitivity and specificity were 0.710 and 0.911, respectively. Three changes in texture parameters were independent factors associated with patient survival, with a hazard of 0.173, 2.068, and 1.940, respectively. The C-index of the OS predicted by the texture features model was not statistically different from that of the mRECIST (0.695 vs. 0.668, p=0.493). While the C-indices of skewness in the portal venous phase combined with mRECIST (0.729, p=0.015), skewness in the delayed phase combined with mRECIST (0.715, p=0.044), and skewness in both two phases combined with mRECIST (0.728, p=0.017) were statistically different.

**Conclusion:**

Changes in the texture parameters of CT images before and after TACE treatment can be used to obtain relevant grayscale histogram parameters for evaluating the early efficacy of TACE in HCC treatment. And the texture analysis combined with mRECIST may be superior to the mRECIST alone in predicting survival in HCC after TACE treatment.

## Introduction

Hepatocellular carcinoma (HCC) is a common tumor that seriously endangers patients’ lives. A large proportion of patients with HCC in China are at an advanced stage at the time of diagnosis and are often accompanied by liver cirrhosis, making radical surgery impossible. Transarterial chemoembolization (TACE)is an important palliative treatment widely used in unresectable HCC because of the high heterogeneity and instability of advanced HCC, the treatment process is complicated, it is often necessary to combine multiple methods for sequential treatment based on individual differences, different responses to treatment, etc. In order to effectively guide individualized treatment to the survival benefits of patients, the treatment strategy needs to be adjusted accurately. The efficacy evaluation of TACE includes two aspects: survival period and imaging. Imaging evaluation mainly depends on the professional level and subjective judgment of diagnostic doctors. These analytical judgments are often subjective, qualitative, or semi-quantitative and lack precise quantification, making it difficult to identify high-risk individuals.

The idea of radiomics is mainly derived from the heterogeneity of tumors, which assumes that microscopic changes in diseases, such as genes and cells, can be translated to imaging characteristics of macroscopic aspects, such as tissues and organs (1, 2). Texture analysis is an important image-feature extraction method. By means of statistics, models, or functions, texture analysis can quantitatively analyze the heterogeneity of lesions on medical images, determine their regularity, and provide assistance for the qualitative diagnosis of lesions, clinical grading and staging of tumors (3), tumor efficacy evaluation (4), and prognosis prediction (5, 6). There have been numerous achievements and wide recognition of this method. Previous studies have found that texture analysis can be used to identify the differentiation (7, 8) and microvascular invasion of HCC (9) before surgery, predict the response to TACE and systemic treatment (10, 11), and predict the prognosis after hepatectomy in HCC patients (12, 13), such as early recurrence (14, 15) and survival time (16). Vandecaveye et al. (17) performed texture analysis on diffusion-weighted MRI (DWI) images in patients with HCC before and after TACE treatment, and the Apparent Diffusion Coefficient (ADC) ratio before and after treatment was found to be an independent predictor of Progression-free survival (PFS), which had a stronger correlation with tumor response. Kloth et al. (18) performed texture analysis on CT images after drug-eluting bead transarterial chemoembolization, which did not involve lipiodol deposition. However, there have been few reports on texture analysis of CT images after traditional lipiodol TACE treatment.

This study aimed to analyze texture parameters of enhanced CT before and after TACE treatment in HCC, and compare them with the Modified Response Evaluation Criteria in Solid Tumors (mRECIST) to determine the value of CT texture analysis in the evaluation of TACE efficacy and survival predication in HCC.

## Materials and methods

### Patients

This study was approved by our institutional review board. Between September 2012 and May 2018, HCC patients hospitalized at the Tongji Hospital, Tongji Medical College, Huazhong University of Science and Technology, who received TACE as primary treatment for the first time were enrolled in this study. Patients were followed-up regularly until death or at the last visit. The patients who were still alive on May 10, 2021, were censored.

The inclusion criteria were as follows (1): clinically or pathologically diagnosed with HCC according to the “Primary Liver Cancer Diagnosis and Treatment Standards” (2017 edition, China); (2) with lesions with a maximum diameter ≥5 cm among the target lesions treated by TACE; and (3) underwent a three-stage dynamic contrast-enhanced CT examination within 4 weeks before and 4–8 weeks after TACE treatment, with a slice thickness of 5 mm.

Exclusion criteria were: (1) no obvious enhancement of HCC on CT arterial-phase images before treatment;(2) distant metastases when HCC was diagnosed, such as lung metastases and bone metastases; (3) the maximum diameters of all target lesions treated by TACE were <5 cm; (4) the patient concurrently received other treatment including systemic medicine, surgical resection, liver transplantation, local ablation therapy (radio frequency, microwave, alcohol, argon-helium knife, and Haifu knife), and radiotherapy; and (5) no contrast-enhanced CT images before or after treatment, poor CT images quality or inconsistent slice thickness.

**Figure 1:**
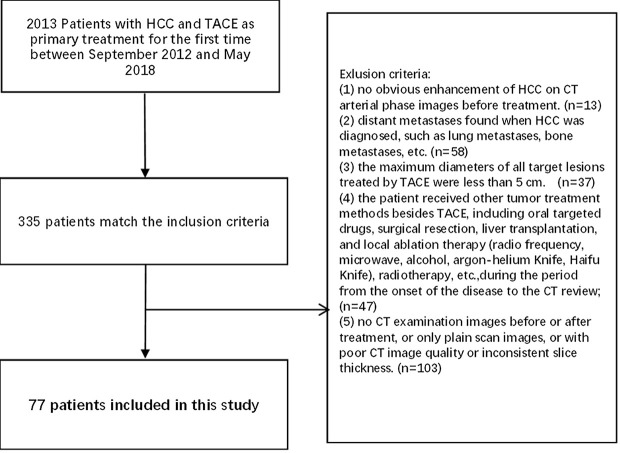
Flowchart of inclusion and exclusion criteria of our cohort.

Finally, 77 HCC patients were enrolled in this study, including 67 males and 10 females, aged 26–81 years, with an average age of 52.73 ± 11.60 years (**Figure 1**).

### CT scan method

All patients signed an informed consent form before the CT scan. After fasting for 4 to 6 h, three-phase enhanced CT scans were performed in a supine position with a scanning coverage from the top of the diaphragm to the inferior border of the liver using a spiral CT scanner (Light Speed VCT; GE Medical Systems, Milwaukee, WI). The scanning parameters were as follows: 120 kV, 250 Ma; slice thickness, 5 mm; slice spacing, 5 mm, matrix 512X512. Lopromide (370 mg I/ml) was injected as a contrast agent using a high-pressure syringe through the cubital vein, and the total amount was calculated according to the formula [height (cm)-100+weight (kg)]/2, followed by a constant saline flush of 25 ml at a flow rate of 3 ml/s. The bolus-tracking technique was used in the abdominal aorta, with a trigger attenuation threshold of 120 HU. When the CT value reached 120Hu, arterial phase scanning was performed immediately, portal venous phase and delayed phase scanning were performed at 30s and 200s respectively, and all three phases were breath-held scans.

### TACE treatment procedures

Patients who underwent TACE did not meet the indications for surgical resection, or the patient and his or her agent were unwilling to undergo surgery. The functional status scores proposed by the Eastern Cooperative Oncology Group (ECOG) were 0–2 points, and the Child-Pugh liver function scores were Grade A or B. Additionally, they had to meet the TACE treatment indications according to the “Primary Liver Cancer Diagnosis and Treatment Standards” (2017 edition, China) and had no distant metastases when HCC was diagnosed, such as lung metastases, bone metastases, etc. All patients provided written informed consent before TACE treatment. TACE procedures were successfully performed by operators with more than 3 years of interventional operation experience and under the guidance of superior physicians with more than 10 years of interventional diagnosis and treatment experience. After routine sterilization of the groin area, spreading of sterile towels, and local anesthesia with 5 mL of 2% lidocaine, the femoral artery was punctured using the modified Seldinger technique. After inserting the catheter sheath, a 5F catheter was inserted for the celiac artery and superior mesenteric artery angiography, using iopromide (370 mg I/ml) at a dose of 15 ml and at a flow rate of 5 ml/s. The tumor-feeding artery was selectively catheterized using a catheter or microcatheter. When the tumor has multiple arteries supplying blood, embolization should be performed individually. An emulsion made of super-liquefied lipiodol and lobaplatin was slowly injected as an embolic agent under Digital subtraction angiography (DSA) fluoroscopic monitoring, with dosages of super-liquefied lipiodol and lobaplatin less than 20 ml and 50 mg, respectively. After the injection, a certain amount of gelatin sponge particles was administered for embolization; their size and dosage were determined by a superior physician.

### Image texture analysis

**Figure 2:**
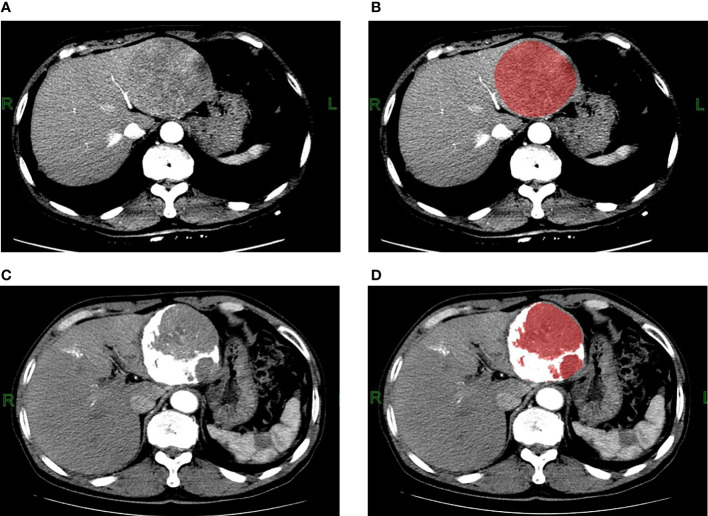
Contrast-enhanced CT arterial phase images and the ROI images before TACE treatment. **(A, B)** A 62-year-old male patient with hepatitis B and small three-yang, AFP greater than 80000ng/L before TACE treatment; three-phase enhanced CT examination 5 days before treatment showed that the lesion was located in the left lobe of the liver, with a maximum cross-section of about 90mm*85mm, no cirrhosis, no portal vein tumor thrombus or arteriovenous fistula, with heterogeneous enhancement in the arterial phase. The ROI was manually delineated using the FireVoxel software, and the grayscale histogram parameters were extracted for texture analysis. Contrast-enhanced CT arterial phase images and ROI images after TACE treatment. **(C, D)** The patient underwent three-phase enhanced CT examination 39 days after TACE treatment. The maximum section of the lesion was about 83mm*77mm, and the TACE treatment effect was evaluated as stable disease SD. After delineating the ROI, the CT value was used to split the ROI, the white area larger than 200Hu was the lipiodol deposition, and the red area smaller than 200Hu was the final ROI, the texture analysis of the final ROI was performed.

To ensure the uniformity of the data, all cases were analyzed and processed using 5 mm slice thickness images. The CT images with 5 mm slice thickness in DICOM format before and after TACE treatment were downloaded through PACS and then imported into the FireVoxel software (NYU Center for Advanced Imaging Innovation and Research, New York, USA) for measurement. The delineation of the ROI was determined by the consensus of two radiologists, both of whom had more than 3 years of experience in abdominal radiology and were blinded to the prognosis. As TACE does not guarantee the simultaneous treatment of all lesions, only the target lesions were segmented and analyzed. Volume mapping was performed at all levels of the lesions, and necrosis and large blood vessels in the lesions were carefully avoided during ROI delineation. For multiple lesions, the largest lesion that was treated with TACE was selected. For the CT images after TACE, the ROIs were split with CT values, and voxels with CT values greater than 200Hu in the ROIs were discarded to eliminate the influence of lipiodol deposition (**Figure 2**). Sixteen texture parameters were calculated for each phase, including the mean, standard deviation, heterogeneity, skewness, kurtosis, entropy, minimum, maximum, median, and 5th, 10th, 25th, 50th, 75th, 90th, and 95th percentiles. All three phases of the CT enhancement images were analyzed. There were 48 texture parameters for all three phases, and 48 changes in texture parameters calculated as (post-treatment - pre-treatment parameters). The maximum, minimum, mean, median, and all percentile values are the CT values of the grayscale histogram. In the FireVoxel software calculation, 1024Hu was added based on the original CT value. All statistical charts in the results of this study are values obtained after adding 1024.

### Patients grouped according to the treatment’s efficacy

The treatment response to TACE was determined independently by two radiologists according to the mRECIST, both of whom had more than three years of experience in abdominal radiology. A radiologist with more than 10 years of experience in abdominal radiology participated in the consultation if there were inconsistent results, and the decision was made by consensus. None of the three radiologists were involved in the ROI delineation process and were blinded to the patient’s prognosis. The clinical manifestations and alpha-fetoprotein (AFP) levels were only used as references. Patients with complete and partial responses were in the remission group, and those with stable disease and progressive disease were in the non-remission group.

### Statistical analysis

Statistical analysis was performed using SPSS23.0 software (SPSS, Inc, Chicago, IL) and R software (version 3.6.1, http://www.r-project.org/). The data were tested for normality using the Shapiro-Wilk test. Data that obeyed a Gaussian distribution are presented as mean ± standard deviation, and non-Gaussian distribution data are presented as the median and interquartile range. Only when the data of both remission and non-remission groups obeyed the Gaussian distribution, the independent-sample t-test were used, and the corresponding p- value was selected according to the results of the Levene’s test for homogeneity of variance; otherwise, the Mann-Whitney U test was performed. For enumeration data, statistical differences were compared using the chi-squared test. Statistical significance was set at p < 0.05. For parameters with statistical significance, the ROC curve was used to analyze the diagnostic performance, and the corresponding AUC and maximum Youden index were calculated. The survival of all patients was followed, and the date of death was recorded for each patient. The follow-up period ended on May 10, 2021. The Kaplan-Meier method was used to compare survival between the remission and non-remission groups. A multifactorial Cox proportional-hazards model (also known as Cox regression) was used to test whether the differences in the 48 grayscale histogram parameters in the three phases were statistically significant in predicting survival. The C-index of the mRECIST-predicted OS was calculated, and texture features with predictive values were screened using a multifactorial Cox model to construct a prediction model. The C-index of OS was predicted using the individual texture parameters with predictive value and the prediction model, and compared with the C-index of the mRECIST-predicted OS (**Table 4**). The C-index between the different models was compared using the “compare C” package in R. It is based on a nonparametric analytical approach and compares two C-indexes using a z-test (19).

## Results

### General characteristics of patients before TACE treatment

The clinical characteristics of the patients were summarized in **Table 1**. According to the mRECIST, the treatment response was evaluated as follows:12 cases of complete remission (CR), 19 cases of partial remission (PR), 27 cases of stable disease (SD), and 19 cases of progressive disease (PD). Thus, 31 cases were included in the remission group (CR and PR), and 46 cases were included in the non-remission group (SD and PD). The median value of the target lesion volume in the remission group was 102.45 cm³, and the median value of the target lesion volume in the non-remission group was 447.28 cm³. The maximum target lesion in the enrolled cases was 17.0*15.0*11.3 cm³, 2881.50 cm³. There were statistical differences in the volume of the lesions before treatment between the two groups, but no statistical difference was observed in the other data (**Table 1**).

**Table 1:** Comparison of the general characteristics between two groups before TACE treatment.

		Remission Group	Non-remission group	p
**No. of patients**		31	46	
**Gender**	Male	26	41	
	Female	5	5	p>0.05
**Age**		54.16 ± 14.42	51.76 ± 10.91	p>0.05
**Hepatitis status**	No hepatitis	6	10	
	Hepatitis B	25	36	p>0.05
**AFP (ng/mL)**	Negative	8	6	
	Positive, less than 400	9	9	
	Positive, greater than 400	14	31	p>0.05
**Lesion Volume (cm3)**		102.45^*^	447.28^*^	**p<0.05**
**No. of lesions**	Single shot	20	24	
	Multiple shots	11	22	p>0.05
**Portal vein tumor thrombus**	Yes	12	15	
	No	19	31	p>0.05
**Liver cirrhosis**	Yes	13	16	
	No	18	30	p>0.05

*The volume of lesions in both groups did not conform to a normal distribution; therefore, the median was used.

The bold values indicate the corresponding p-values less than 0.05.

### Statistical analysis results of grayscale histogram parameters

A total of 23 changes in parameters pre-and post-TACE (the mean, standard deviation, heterogeneity, skewness of the three phases, entropy, 75th, 90th, and 95th percentile of the portal venous phase and delay phase, and max, 5th, 10th percentile of portal venous phase) were tested using an independent-sample t-test, and the Mann-Whitney U test was applied to the difference values of other parameters. There was a significant difference between remission and non-remission groups in 41 changes pre-and post-TACE, which were: mean, standard deviation, heterogeneity, skewness, kurtosis, median,5th, 10th, 25th, 50th, 75th, 90th, and 95th percentile in the three phases, and entropy in the arterial and delay phases (**Table 2**).

**Table 2:** Differences in three-phase grayscale histogram parameter difference values between remission group and non-remission group.

Difference value (post-front)	Arterial phase			Portal venous phase			Delayed phase		
	Remission Group	Non-remission group	p	Remission Group	Non-remission group	p	Remission group	Non-remission group	p
Mean	46.66 ± 24.48	18.23 ± 16.02	**0.000**	41.05 ± 20.46	19.07 ± 15.18	**0.000**	45.19 ± 22.53	20.38 ± 15.64	**0.000**
Standard deviation	20.10 ± 7.80	13.20 ± 7.80	**0.000**	16.87 ± 7.27	11.76 ± 7.36	**0.004**	20.59 ± 6.98	14.23 ± 8.22	**0.000**
Heterogeneity	0.02 ± 0.01	0.01 ± 0.01	**0.001**	0.01 ± 0.01	0.01 ± 0.01	**0.010**	0.02 ± 0.01	0.01 ± 0.01	**0.002**
Skewness	0.15 ± 1.09	0.97 ± 0.52	**0.000**	0.44 ± 0.70	0.95 ± 0.46	**0.000**	0.67 ± 0.77	1.35 ± 0.46	**0.000**
Kurtosis	-0.67	1.42	**0.000**	-0.11	1.04	**0.000**	-0.25	1.83	**0.000**
Entropy	0.49	0.24 ± 0.28	**0.000**	0.22 ± 0.39	0.14 ± 0.21	0.305	0.28 ± 0.33	0.08 ± 0.30	**0.008**
Minimum	-0.84 ± 54.66	-6.00	0.105	-4.00	-8.00	0.401	-3.00	-16.00	0.126
Maximum	53.00	37.00	0.962	50.61 ± 31.34	50.84 ± 32.16	0.975	75.35 ± 18.03	81.00	0.977
Median	36.00	13.09 ± 15.26	**0.000**	37.13 ± 23.09	14.00	**0.000**	36.00	14.73 ± 14.53	**0.000**
5th percentile	13.00	6.00 ± 9.76	**0.004**	19.55 ± 18.29	7.27 ± 9.37	**0.001**	12.00	7.20 ± 8.99	**0.003**
10th percentile	15.00	7.13 ± 10.35	**0.001**	22.05 ± 18.73	9.33 ± 9.81	**0.001**	18.00	8.59 ± 9.20	**0.000**
25th percentile	22.00	9.76 ± 11.90	**0.000**	27.94 ± 20.04	10.00	**0.000**	24.00	10.50 ± 10.60	**0.000**
50th percentile	37.00	13.09 ± 14.66	**0.000**	36.84 ± 23.31	14.00	**0.000**	36.00	14.11 ± 15.23	**0.000**
75th percentile	59.98 ± 32.08	16.00	**0.000**	51.68 ± 26.09	22.71 ± 19.53	**0.000**	57.58 ± 28.98	23.23 ± 21.56	**0.000**
90th percentile	82.00	36.69 ± 28.28	**0.000**	66.81 ± 25.64	36.13 ± 26.03	**0.000**	76.61 ± 27.44	40.50 ± 28.74	**0.000**
95th percentile	91.00	49.73 ± 31.70	**0.000**	74.29 ± 22.08	47.77 ± 29.29	**0.000**	86.29 ± 23.35	54.64 ± 31.18	**0.000**

There were multiple sets of difference values of the grayscale histogram parameters between the remission group and the non-remission group that did not conform to the normal distribution, so the median was used.

The bold values indicate the corresponding p-values less than 0.05.

### Diagnostic performance of statistically significant grayscale histogram parameters

**Figure 3:**
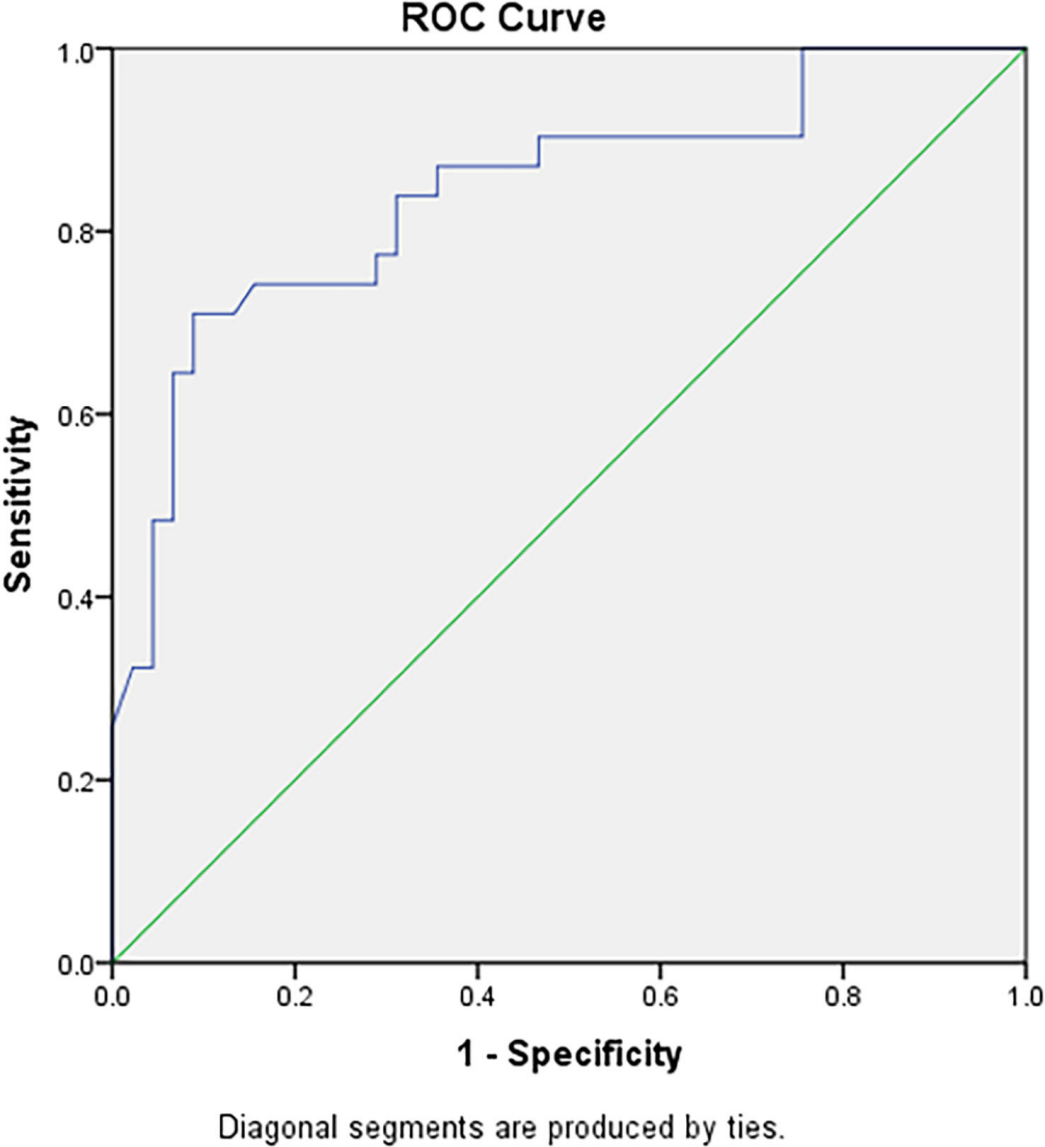
The ROC curve diagnostic efficiency graph based on the difference of the 90th percentile in the arterial phase before and after TACE treatment between the remission group and the non-remission group. It shows an AUC of 0.842, with cutoff value of 70.50, and the largest Youden index (0.621), with a sensitivity and specificity of 0.710 and 0.911 respectively.

The ROC curves of the 41 changes in texture parameters pre- and post-TACE were drawn. According to the AUC of the ROC curves, the top three in the arterial phase were the 90th percentile, 75th percentile, and 95th percentile, and their AUCs were 0.842, 0.837, and 0.828, respectively; the top three in the portal venous phase were the mean, 75th percentile, and median, and the AUCs of their ROC curves were 0.809, 0.807, and 0.800, respectively; the top three in the delayed phase were the 75th percentile, 50th percentile and 90th percentile, and the AUCs of their ROC curves were 0.830, 0.821, and0.821, respectively(**Table 3**). The AUC of the changes in the 90th percentile in the arterial phase pre- and post-TACE was the largest (0.842). When the cut-off value was 70.50, the Youden index was the largest (0.621), and the sensitivity and specificity were 0.710 and 0.911, respectively (**Figure 3**).

**Table 3:** Diagnostic performance of statistically significant grayscale histogram parameters.

Phases	difference value (post-front)	AUC	95% CI	Cut-off	YI	Sensitivity	Specificity
**Arterial phase**							
	Mean	0.825	0.725-0.924	34.098	0.576	0.709	0.866
	Standard deviation	0.724	0.608-0.839	22.530	0.352	0.419	0.933
	Inhomogenity	0.703	0.584-0.823	0.019	0.352	0.419	0.933
	Skewness	0.787	0.675-0.899	0.911	0.515	0.870	0.644
	Kurtosis	0.822	0.713-0.931	0.686	0.626	0.870	0.755
	Entropy	0.741	0.624-0.859	0.417	0.497	0.741	0.755
	Median	0.805	0.701-0.908	18.000	0.493	0.870	0.622
	5th	0.693	0.568-0.818	19.000	0.374	0.419	0.955
	10th	0.731	0.615-0.848	25.000	0.387	0.387	1.000
	25th	0.787	0.680-0.893	17.500	0.475	0.741	0.733
	50th	0.809	0.707-0.912	35.000	0.503	0.548	0.955
	75th	0.837	0.740-0.934	43.500	0.566	0.677	0.888
	90th	0.842	0.747-0.936	70.500	0.620	0.709	0.911
	95th	0.828	0.733-0.923	86.500	0.576	0.709	0.866
**Portal venous phase**							
	Mean	0.809	0.705-0.912	26.988	0.551	0.774	0.777
	StDev	0.680	0.556-0.804	16.443	0.336	0.580	0.755
	Inhomogenity	0.653	0.526-0.780	0.011	0.319	0.741	0.577
	Skewness	0.755	0.638-0.872	0.455	0.501	0.612	0.888
	Kurtosis	0.759	0.639-0.880	0.477	0.463	0.774	0.688
	Median	0.800	0.692-0.907	21.500	0.541	0.741	0.800
	5th	0.723	0.602-0.844	12.500	0.390	0.612	0.777
	10th	0.729	0.608-0.850	12.500	0.408	0.741	0.666
	25th	0.769	0.654-0.883	15.500	0.517	0.806	0.711
	50th	0.793	0.684-0.902	19.500	0.539	0.806	0.733
	75th	0.807	0.702-0.912	38.000	0.596	0.774	0.822
	90th	0.791	0.683-0.898	56.500	0.574	0.774	0.800
	95th	0.758	0.648-0.867	64.500	0.507	0.774	0.733
**Delay** **phase**							
	Mean	0.817	0.720-0.914	31.588	0.546	0.774	0.772
	StDev	0.727	0.610-0.844	19.808	0.408	0.612	0.795
	Inhomogenity	0.708	0.587-0.828	0.017	0.408	0.612	0.795
	Skewness	0.816	0.707-0.926	1.016	0.615	0.774	0.840
	Kurtosis	0.787	0.679-0.895	0.348	0.537	0.741	0.795
	Entropy	0.653	0.526-0.781	0.303	0.302	0.483	0.818
	Median	0.820	0.722-0.917	23.500	0.524	0.774	0.750
	5th	0.697	0.575-0.819	18.500	0.328	0.419	0.909
	10th	0.754	0.640-0.868	8.500	0.416	0.870	0.545
	25th	0.788	0.681-0.894	15.000	0.456	0.774	0.681
	50th	0.821	0.725-0.918	23.500	0.524	0.774	0.750
	75th	0.830	0.735-0.925	36.000	0.556	0.806	0.750
	90th	0.821	0.723-0.918	59.000	0.533	0.806	0.727
	95th	0.795	0.694-0.897	75.500	0.501	0.774	0.727

### Effectiveness of grayscale histogram parameters in predicting survival

**Figure 4:**
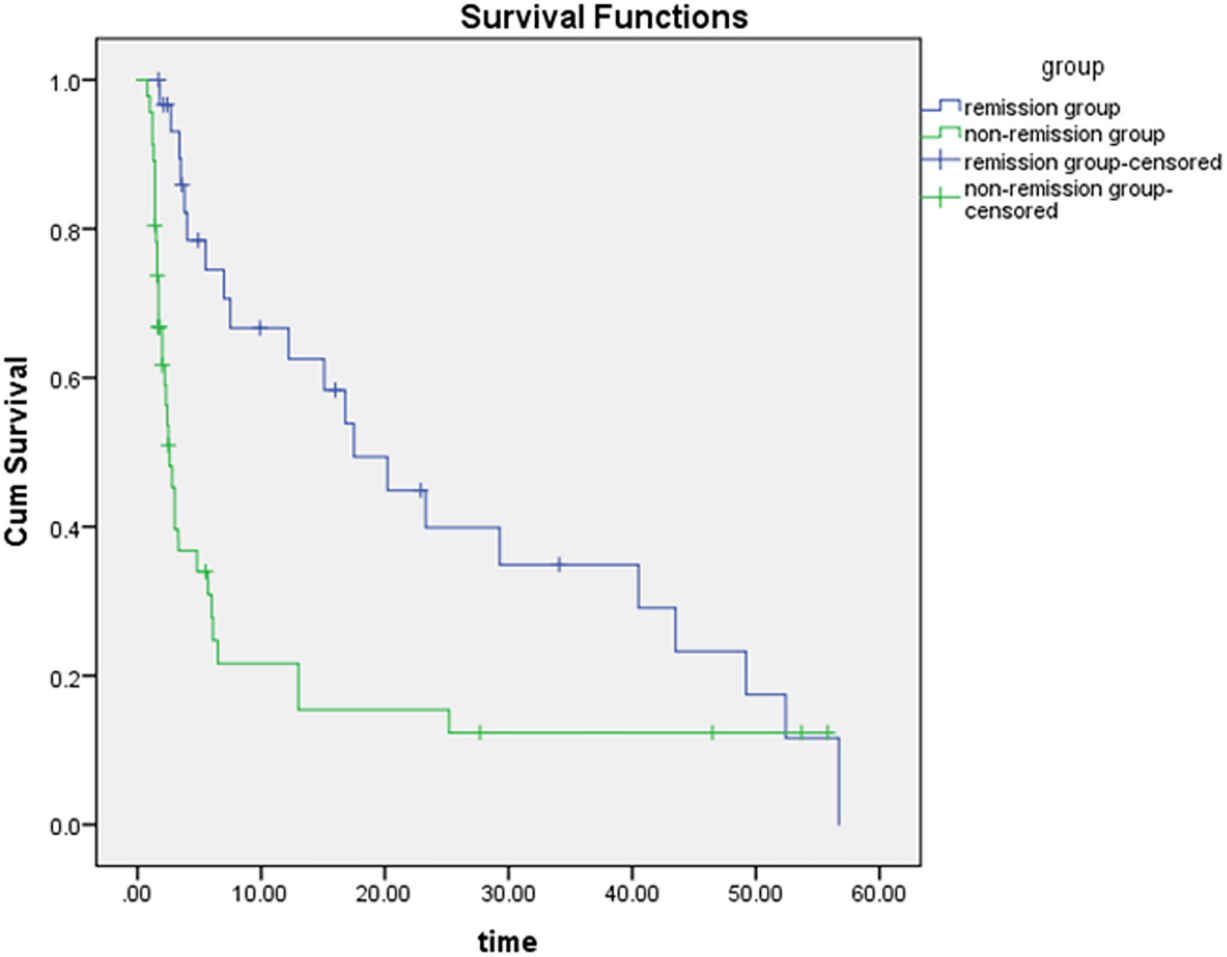
The difference in survival time between the two groups is statistically significant by the Kaplan-Meier method (p = 0.000), the dark blue curve is the survival curve of the remission group and the green curve is the survival curve of the non-remission group.

The median survival was 17.50 months in the remission group and 2.60 months in the non-remission group. The difference in survival between the two groups was statistically significant using the Kaplan-Meier method (**Figure 4**; CR and PR in the remission group were group 1, dark blue; and SD and PD in the non-remission group were group 2, green). The results of the multivariate Cox proportional-hazards model showed that the change in entropy in the arterial phase (p = 0.000), the change in skewness in the portal venous phase (p = 0.001), and the delayed phase (p = 0.001) were independent factors associated with patient survival, with hazard ratios (HR) of 0.173, 2.068, and 1.940, respectively, and corresponding 95% confidence intervals (CI) were 0.069-0.435, 1.363-3.135 and 1.292-2.913, respectively. Changes in the other 45 grayscale histogram parameters were not statistically significant in predicting survival. The C-index of OS predicted by the mRECIST was 0.668. Among the differences in texture parameters, the C-index of OS predicted by entropy in the arterial phase, skewness in the portal venous phase, skewness in the delayed phase, and the texture features model were 0.666, 0.680, 0.675, and 0.695, respectively, which were not statistically different from the C-index of OS predicted by the mRECIST. The C-indices of OS predicted by skewness in the portal venous phase combined with mRECIST (0.729, p=0.015), skewness in the delayed phase combined with mRECIST (0.715, p=0.044), and skewness in both two phases combined with mRECIST (0.728, p=0.017) were statistically different. (**Table 4**).

**Table 4:** C-index comparison of the mRECIST, texture parameter difference values with predictive value, texture features model and texture features combined with mRECIST in predicting OS.

		C-INDEX	95%CI		p
			Low	High	
**mRECIST**		0.668	0.614	0.721	
**difference value (post-front)**					
	Arterial phase-entropy	0.666	0.587	0.746	0.975
	Portal venous phase-skewness	0.680	0.595	0.766	0.771
	Delay phase-skewness	0.675	0.596	0.755	0.840
**texture features model**		0.695	0.622	0.768	0.493
**texture features combined with mRECIST**	A-entropy + mRECIST	0.710	0.642	0.777	0.176
	P-skewness + mRECIST	0.729	0.657	0.801	**0.015**
	D-skewness + mRECIST	0.715	0.646	0.785	**0.044**
	A-entropy + P-skewness + mRECIST	0.722	0.656	0.788	0.058
	A-entropy + D-skewness + mRECIST	0.716	0.653	0.780	0.102
	P-skewness + D-skewness + mRECIST	0.728	0.656	0.800	**0.017**
	A-entropy + P-skewness + D-skewness + mRECIST	0.721	0.655	0.787	0.062

The p-value was calculated based on the statistical comparison of these texture parameters with the mRECIST.

The bold values indicate the corresponding p-values less than 0.05.

## Discussion

TACE is the main method for non-surgical treatment of HCC, currently recognized as one of the most common methods for non-surgical treatment of liver cancer, and is widely used in palliative treatment before liver transplantation (20), down-stage treatment of unresectable HCC, adjuvant treatment after surgical resection, etc. However, residue, recurrence, and metastasis often occur after TACE, and it is necessary to combine multiple methods for sequential treatment based on individual response (21–24). It is of great significance to accurately evaluate the short-term efficacy of TACE and adjust the treatment strategy in a timely manner to effectively improve the survival of the patients. Short-term evaluation of TACE is closely related to imaging evaluation. However, the traditional imaging evaluation of efficacy is usually based on density, size, shape, boundary, enhancement features, secondary changes, etc., and the information judgment is closely related to the radiologist’s experience. Many signs are subjective, non-quantitative, or only semi-quantitative and lack precise quantification, making it difficult to identify high-risk individuals.

Our study aimed to perform a texture analysis of CT images before and after TACE treatment and to calculate the changes in texture parameters when compared with traditional efficacy evaluation methods. We found that 41 changes in the texture parameters were statistically significant between the remission and non-remission groups. Among the 41 statistically significant changes, the diagnostic efficacy of the 90th percentile difference value in the arterial phase (AUC of the ROC curve) reached 0.842, which was related to the selection of the mRECIST standard for efficacy evaluation in this study. Unlike previous evaluation criteria, the mRECIST criteria do not focus on changes in lesion volume with the largest cross-section or maximum diameter, but on the sum of the diameters of enhanced lesions in the arterial phase. Early studies (17, 18) suggested that because texture parameters could quantify image characteristics, the changes in texture parameters before and after treatment may reflect the changes in tumor heterogeneity to some extent, and the corresponding form may not be a simple functional form. In this study, we attempted to evaluate the therapeutic effect with CT texture parameters after TACE treatment, but the effect was not good; we then attempted to calculate the changes in the texture parameters of the CT images before and after TACE treatment. Comparing the difference and ratio values of the texture parameters before and after treatment between the remission and non-remission groups, it was found that there were more statistically significant texture parameters in the difference values, and the corresponding AUCs of the ROC curves were also larger. Therefore, we chose the difference value as the research object in the results of this study.

The imaging evaluation criteria mainly included RECIST, mRECIST, EASL, WHO standards. Several studies have compared the advantages and disadvantages of these criteria in TACE (25–30), and the majority of them showed that the mRECIST criteria are relatively highly consistent and easy to operate. However, only arterial phase enhancement was evaluated, and the assessment of lesions without arterial phase enhancement was not accurate. Because the mRECIST standards were used and required as accurately as possible, patients whose lesions had no arterial phase enhancement before treatment were excluded from our study.

In the survival-based assessment, among the 48 grayscale histogram parameters in the three phases, the change in entropy in the arterial phase, skewness in the portal venous phase, and delayed phase were independent factors associated with patient survival. Although there was no statistical difference between the texture parameter and texture parameter model predicted OS compared to the mRECIST, texture analysis could be a robust way to predict the efficacy of TACE treatment, which is mostly independent from readers and has less interobserver variability. Additionally, the C-indices of skewness in the portal venous phase, in the delayed phase, and in both two phases combined with mRECIST were statistically different. This indicates that texture analysis combined with mRECIST may be superior to the mRECIST alone in predicting survival.

In this study, CT was selected as the imaging evaluation tool and texture analysis material to assess TACE efficacy. MRI has unique advantages in identifying necrotic tissues and residual recurrence of lesions, including the application of DWI (17), MRS (31, 32), BOLD (33), and other technologies, which is also conducive to a clear diagnosis, but it is more expensive and cannot evaluate lipiodol deposition. Although the deposition of lipiodol is not directly related to its efficacy, it is of great value in clinical diagnosis and development of follow-up treatment plans. CT is more commonly used in the whole process of TACE treatment of HCC, and has many advantages, such as: measuring lesion volume and liver volume, displaying tumor blood supply artery morphology and variation, three-dimensional reconstruction to find ectopic blood supply, discovering hepatic artery-portal vein or hepatic vein fistula, evaluation of tumor bone and lung metastasis, and observation of lipiodol deposition (34–38). The short appointment time and few restrictions on examination also give CT examination unique advantages in the follow-up review of lipiodol TACE. However, CT examination is affected by lipiodol deposition and the volume effect of the high-density part of lipiodol, which may lead to the missed diagnosis of residual lesions or recurrence.

Image segmentation methods include automatic, semi-automatic, and manual methods. The objectivity of the automatic segmentation technique is superior to that of manual segmentation; automatic segmentation cannot outline the lesion completely correctly for the time being. Manual segmentation is generally considered the gold standard, although the workload is large, time-consuming, and labor-intensive, and the interference of subjective factors cannot be completely avoided. Manual segmentation of the ROI was used in this study, and subjective factors were avoided: first, only HCC lesions with volumes (the maximum diameter of the target lesion ≥ 5 cm) were selected; second, some parts of the marginal area of the lesions that could not be identified as lesions were not included. This approach was inspired by the study of Echegaray et al. (39), who found that the maximum inscribed circle method had a good correlation with manual segmentation of the lesion, and we speculated that ROI segmentation did not require complete coverage. Third, radiologists were blinded to the efficacy. In addition, Ng, Francesca et al. (40) believed that texture analysis of full volume data may be more representative of tumor heterogeneity; therefore, this study chose full volume data for texture analysis instead of using the maximum area section method or multiple section method.

In this study, we performed some experimental explorations for processing lipiodol deposition in CT images after c-TACE and finally chose to eliminate the effect of lipiodol deposition. Although the morphology of lipiodol deposition and its proportion to the volume of the lesion can provide valuable diagnostic information (34), as the CT value of lipiodol is much higher than that of the lesion, high-density lipiodol affects many of the grayscale histogram parameters when the lipiodol and the lesion are subjected to texture analysis at the same time. The deposition of lipiodol may be flakes or scattered spots, and it is difficult to distinguish lipiodol deposition from lesion enhancement and blood vessels with the naked eye, making it difficult to manually remove lipiodol deposition when delineating the ROIs. To eliminate the influence of lipiodol deposition on the texture parameters of the lesions, this study analyzed voxels with CT values lower than 200 HU in the ROIs and discarded those with CT values higher than 200 HU. We found that the 200HU CT value would be better than other values as the limit, but whether there is a better method to eliminate the lipiodol deposition needs further exploration and research.

This study has some limitations. First, the texture parameters include first-order, second-order, and high-order parameters, and this study only extracted the first-order parameters. The extraction of second-order and high-order parameters and the combination with related clinical indicators are expected to improve diagnostic efficiency in future research. Second, some studies have shown that thin-slice images are more suitable for texture analysis than thick-slice images (41); however, because of the workload of manual segmentation and the lack of thin-slice images for some early cases, this study chose 5 mm slice thickness images.

In conclusion, texture analysis of contrast-enhanced CT before and after TACE treatment could help evaluate the efficacy of TACE in HCC treatment. And the texture analysis combined with mRECIST may be superior to the mRECIST alone in predicting survival in HCC after TACE treatment.

## Data availability statement

The raw data supporting the conclusions of this article will be made available by the authors, without undue reservation.

## Ethics statement

The studies involving human participants were reviewed and approved by the Medical Ethics Committee at Tongji Medical College, Huazhong University of Science and Technology. Written informed consent for participation was not required for this study in accordance with the national legislation and the institutional requirements.

## Author contributions

YL: study design. YL and XH: study conduct. HZ and YH: clinical data collection and processing. WZ and JL: statistical analysis. YL and YL: CT reading. WZ: drafting manuscript. All authors contributed to the article and approved the submitted version.

## Funding

This work was supported by the grants from National Natural Science Foundation of China (NSFC) No. 82001786.

## Conflict of interest

The authors declare that the research was conducted in the absence of any commercial or financial relationships that could be construed as a potential conflict of interest.

## Publisher’s note

All claims expressed in this article are solely those of the authors and do not necessarily represent those of their affiliated organizations, or those of the publisher, the editors and the reviewers. Any product that may be evaluated in this article, or claim that may be made by its manufacturer, is not guaranteed or endorsed by the publisher.
